# Accidents and Apathy: The Construction of the ‘Robens Philosophy’ of Occupational Safety and Health Regulation in Britain, 1961–1974

**DOI:** 10.1093/shm/hkv068

**Published:** 2015-06-24

**Authors:** Christopher Sirrs

**Keywords:** regulation, accidents, occupational health and safety, Robens, Britain

## Abstract

The 1972 Robens Report is widely regarded to have provided the underlying rationale for the ‘modern’ system of occupational health and safety regulation in Britain, embodied in the Health and Safety at Work Act (HSW Act) 1974. The HSW Act advanced a new, more flexible system of regulation, premised on the ideal of self-regulation by industry. This article advances a more nuanced historical understanding of the Report and its ethos—the ‘Robens philosophy’—than hitherto developed, situating its assumptions about accidents, regulation and the role of the state in the social, economic and political context of Britain in the 1960s and early 1970s. Highlighting the interaction between these trends and long-established regulatory practices, the article argues that the turn to ‘self-regulation’ heralded by the Robens Report was highly convincing from a political and regulatory perspective at the time it was promulgated.

## Introduction

In the early 1970s, Britain's accident record left much to be desired for factory inspectors, trade unionists, safety charities and other bodies concerned about safety at work. Despite a significant fall in fatal accidents in factories, workshops and other premises since the beginning of the twentieth century, by the 1970s there were growing concerns both in and outside government that the existing approach to regulating workplace safety, laid down in the nineteenth century, had run out of steam.^1^ Around 1,000 people each year were being killed as a result of work, and half a million more were being injured—the true figure was probably much higher than this due to the recognised problem of under-reporting. The government estimated that Britain's accident problem cost the nation some 23 million lost working days a year, or £200 million: a notable sum in the context of a struggling economy seen to be lagging behind its major competitors, West Germany and the USA.^2^

From 1959, the number of reported accidents under the Factories Act, one of the major statutes governing workers' safety, steadily increased. The crisis of faith which enveloped British occupational safety and health regulation motivated the 1964–70 Labour government to appoint an independent Committee on Safety and Health at Work in May 1970, headed by the former chairman of the nationalised coal industry, Lord Alfred Robens (hereafter referred to as the Robens Committee). Charged with reviewing the existing statutory and voluntary arrangements for worker protection, the Committee controversially concluded in 1972 that ‘apathy’ by employers and employees was primarily to blame for workplace accidents.^3^ The Committee believed that excessive or overly detailed regulation could actually promote apathy, by encouraging employers and workers to relinquish their responsibilities, and think that safety and health was a matter for the government. This assertion underpinned the Committee's main recommendation, which was that overall responsibility for regulating safety and health should be rebalanced, with employers and workers themselves assuming the burden.^4^ The Committee's argument that ‘a more effectively self-regulating system’ was needed formed the basis of the Health and Safety at Work etc. Act (HSW Act), passed in July 1974.^5^

Remaining on the statute book to this day, over 40 years later, the HSW Act forms the core of a system that puts voluntary effort, or ‘self-regulation’ by employers and workers at the heart of accident prevention. Codes of practice, voluntary standards and non-statutory forms of guidance are used in preference to detailed, prescriptive regulation to promote a safe, hygienic work environment. Written in ‘goal-based’ terms, the law defines the overall objectives to be achieved, and gives duty holders considerable flexibility in how they comply. Employers are encouraged to evaluate for themselves the steps needed to comply with the law, an obligation that extends to the need to carry out written risk assessments. Employers and trade unions also play a primary role in developing new standards: in 1974 they became represented on a major new body, the Health and Safety Commission (HSC), which performed regulatory and policy-making functions previously carried out by government departments. Under the post-1974 framework, the role of the state is to support the conditions that allow self-regulation to be effective, for instance, by providing technical advice to the HSC and stepping in to prosecute employers who have failed to comply with the Act.^6^

Debates about regulation and the nature of the accident problem in the 1960s and early 1970s thus continue to frame the way we conceptualise and address workplace hazards in Britain today. Yet, to date, the rationale underpinning this influential shift in safety and health regulation, the so-called ‘Robens philosophy’, remains to be given dedicated scholarly attention. Focusing on the response to workplace accidents in the British government in the 1960s and early 1970s, in association with trade unions, employers' associations and safety charities, this article offers the first detailed account of the developments in regulatory thinking and practice that underpinned this pivotal transformation. Drawing upon published and archival material belonging to key actors, including the Ministry of Labour (MOL), Trades Union Congress (TUC) and the Robens Committee itself, the article explores why the Robens Committee felt the need at this juncture to emphasise a turn to greater self-regulation and non-prescriptive law, rather than more detailed regulation or more vigorous enforcement of the existing legal provisions.^7^

Throughout the article, the focus of the analysis is the conditions that shaped the shift in form of regulation, rather than Robens' related proposals regarding its scope, content and institutional machinery. Emphasis is also placed on ‘safety’ rather than ‘health’, reflecting the contemporary preoccupations of the Robens Report and bias of the British government. As one MOL official claimed in 1960: ‘In practice we classify the work as “safety, health and welfare”, which is a more realistic appraisal of its balance, both from the official and industrial point of view.’^8^ However, it should be noted that Robens' arguments relating to occupational health and medicine closely followed his prescriptions on self-regulation and the readjustment of statutory effort.

## A Contentious Logic

This article is not the first to use the term ‘Robens philosophy’. Since 1974, the term has been widely used to refer to the complex of assumptions and beliefs underlying the Robens Committee's recommendations; lately, socio-legal theorists such as Steve Tombs and Robert Baldwin have popularised the term in their analyses of the British health and safety system, an approach that has been adopted in several other countries, including Canada.^9^ However, while the impact of the Robens philosophy on the subsequent shape and success of British health and safety regulation has been much discussed and criticised by these theorists, to date there has been little focus on the wider historical conditions that shaped the philosophy's own development: the factors that supported a greater focus on voluntarism and ‘self-regulation’ at this moment, as opposed to greater statutory intervention or stronger enforcement. The Committee's views have largely been interpreted in isolation.

Criticisms of the Robens philosophy have centred on the Committee's alleged ‘mistaken analysis’ that resulted in it adopting faulty or naive assumptions about regulation.^10^ Baldwin, for example, has argued how the Committee's ‘consensual’ approach, or desire to seek proactive cooperation between employers and workers in accident prevention, ‘resulted in a distorted view of rules and enforcement—almost a starry-eyed one.’^11^ Robens' assumption that there was a natural ‘identity of interest’ between employers and workers in eliminating accidents was challenged by contemporary critics such as Patrick Kinnersly, who argued that it was ‘a dangerous myth which dovetails with the fiction that most accidents are caused by carelessness and can therefore be eliminated if everyone “pulls together”’.^12^

An associated view is that by embedding these assumptions into regulatory practice, the Robens philosophy served to weaken British health and safety law. Tombs and Whyte have argued that the Robens philosophy provided the blueprint for deregulatory efforts by the British government in the 1980s and 1990s.^13^ Robens' emphasis on consensus and ‘tripartism’ (the involvement of government, industry and trade union representatives) to promulgate health and safety policy, they contend, exposed the system to ‘regulatory degradation’. This is because if one of the key ‘partners’ fails to fulfil its obligations—for example, if budgetary cuts mean that the HSE fails to properly enforce health and safety in the workplace—then Robens' self-regulatory model collapses. Similarly, Matthias Beck and Charles Woolfson have highlighted the rhetorical parallels between the Robens philosophy and deregulatory agenda of New Labour.^14^ What is clear is that these socio-legal scholars have been motivated by a normative concern about what health and safety regulation should be like, or health and safety as an example of government regulation, as opposed to an historical concern about how and why the philosophy emerged and took hold in British regulatory practice.

Here also, consensus and tripartism are often seen to have undermined the safety and health of workers, rather than promote it. David Marsh and Wyn Grant have argued that on the basis of deteriorating relations between the TUC, the CBI and the government in the development of economic policy in the late 1960s and 1970s, ‘there can be little confidence that a genuinely tripartite system exist[ed]’.^15^ In contrast, several socio-legal studies have emphasised the relative harmony of tripartite relations in safety and health, in comparison to other areas of industrial relations and countries such as the USA.^16^ As Sandra Dawson *et al.* note, safety and health represented ‘a relatively stable framework of … law across an economically and politically turbulent period.’^17^

Among historians, criticism of the Robens philosophy is more recent, corresponding to the growing interest in occupational health, illness and disease over the past decade. Arthur McIvor has highlighted the Report's emphasis on ‘apathy’ as a strategy for ‘blaming the victim’ (i.e. the worker) for industrial accidents, an argument that takes its cue from Kinnersly and a critical thread of expert commentary dating back to the Report's publication.^18^ Health and safety being a highly politicised field of inquiry, linked to concerns about employment rights and workers' compensation, these commentaries have often focused on the Committee's treatment of workers to the exclusion of its related focus on management. The Robens Committee found management to be equally if not more culpable for accidents, setting the behavioural norm for the entire organisation.^19^

Whether or not the Robens philosophy is valid, conceptually or in practice, it is necessary to understand how and why the philosophy emerged and gained currency if we are to understand how British health and safety regulation developed after 1974. However, the post-1974 history of health and safety regulation has yet to receive significant attention from historians, despite the growing interest in health and safety in general.^20^ Historians have continued to preoccupy themselves with efforts to prevent accidents and diseases in particular industries in the nineteenth and early twentieth centuries, and, following an appeal first made in Paul Weindling's 1985 edited volume, *The Social History of Occupational Health*, have emphasised the experiences and agency of workers.^21^ The wider context of regulation, apart from particular interventions in these sectors, has been largely ignored. There are several possible reasons for this neglect, including that the prevailing social-historical approach has moved away from simplistic legislative or institutional histories in favour of more nuanced analyses focusing on the contestation of accidents and disease at particular workplaces.^22^

A focus on health and safety at the national or systemic level can be equally as nuanced if one focuses on the wider historical contingencies that promoted one particular approach above another. The following account highlights how, at the time the Robens Committee published its findings in 1972, a turn to greater voluntarism and self-regulation was most convincing from a political and regulatory perspective, seemingly being both conceptually sound and pragmatic to implement. Economic and political developments over the 1960s provided fertile ground for a regulatory and political reaction against prescriptive regulation as a tool for promoting workplace safety, and reinforced certain conceptual biases inherent in the pre-1974 enforcement of health and safety law. By the end of the 1960s, they encouraged the British government to undertake a fundamental reappraisal of its role in regulating workplace safety.

## British Health and Safety Regulation before 1974

Prior to the HSW Act, which extended health and safety protection to virtually all workers, British health and safety legislation was highly fragmented. Nine acts and over 500 regulations governed the safety, health and welfare of workers in particular industries and occupations, and extended control over specific hazards, such as ionising radiation. However, while some workplaces, such as chemical plants, were subject to multiple and often conflicting requirements, others, such as hospitals and schools, were excluded from legal coverage altogether.^23^ Some 8 million British workers, almost a third of the entire working population, received no statutory protection from accidents and illnesses resulting from work.^24^

This complex web of law followed a legal template set in the early nineteenth century. The precedent for statutory intervention in working conditions was set not by a concern about the safety and health of workers per se, but by the working conditions of pauper apprentices in textile mills. From 1802, protective legislation expanded across a widening sphere of industrial activity, drawing in more and more industrial workers. In the process, it became more detailed.^25^ The first ‘safety’ provisions, requiring the fencing of dangerous machinery, appeared in the 1844 Factory Act; towards the end of the nineteenth century, legislation expanded to encompass the health of workers in the ‘dangerous trades’, such as lead smelting.^26^

One of the contributing factors towards this fragmentation was the reactivity of British law. According to the economist Sidney Webb, health and safety legislation was ‘a typical example of English practical empiricism’, considering it proceeded from ‘no abstract theory of social justice or the rights of man’ (unlike continental Napoleonic law), but instead responded pragmatically to particular problems as they emerged.^27^ Industrial disasters, for instance, frequently caught political and public attention and were swiftly followed by remedial legislation: as late as 1969, the Mines and Quarries (Tips) Act advanced new legal requirements for colliery spill tips, following the catastrophic landslide at Aberfan, South Wales in 1966 which killed 144 people.^28^

This piecemeal extension of legislation contributed to several legal and administrative difficulties which, by the 1960s, undermined regulation at the level of the workplace, industry and national policy. Many of the laws on the statute book were vague and confusing, difficult for civil servants to unravel, let alone employers.^29^ Others were hopelessly out of date and reflected industrial circumstances that had long since passed. Further, with several inspectorates in existence, each concentrating on a particular industry or class of hazard, administrative conflicts emerged where it was difficult to tell which Act or which inspectorate had precedence.^30^

The largest and oldest of the inspectorates in existence before 1974 was the Factory Inspectorate, founded in 1833 as part of the Home Office. Of particular importance to the Robens philosophy was the ‘conciliatory’ approach of these inspectors towards enforcement. Peter Bartrip has shown that while the first factory inspectors had significant legal powers by twenty-first-century standards, including the ability to enter premises at any time, their readiness to prosecute declined over the nineteenth century. They fell back on a principle of ‘negotiated compliance’, whereby inspectors persuaded employers to meet legal requirements, rather than coercing them. This was driven by several concerns: the need to efficiently allocate scarce resources; an unwillingness to prosecute middle-class businessmen, many of whom were also magistrates; and the location of ‘a conflict-oriented inspectorate operating within a consensus-oriented department’—the Home Office being more amenable to ‘passivity, quietism and accommodation’.^31^ The socio-legal scholar W. Carson has noted that the very existence of the Factory Inspectorate as a statutory enforcement body, separate from the police, acted to ‘conventionalise’ factory crime, suggesting it was a lesser order of criminal offence.^32^ Whatever the reasons behind this reluctance to prosecute, the principle of ‘negotiated compliance’ remained alive and well in the early 1970s. As the Chief Inspector, John Plumbe explained in 1970, prosecution could be counter-constructive if over-rigorously applied:

It is no more thinkable that there should be so many Inspectors that one could be permanently stationed at every works than that, say, every fifth motor car should be a police car to enforce the Road Traffic Acts. … If a situation ever arose in which the Inspectorate were to attempt rigid enforcement of everything that could be driven through the Courts, so that industry ceased to turn to it for advice and guidance, the standards of safety, health and welfare set over the years in the great majority of workplaces would indeed suffer.^33^

Thus, advice and persuasion continued to be the dominant instruments by which the British state regulated health and safety well into the latter half of the twentieth century. In an extensive monograph on regulatory practice, the socio-legal scholar Keith Hawkins has shown how this philosophy persists under the present-day HSE.^34^

Voluntary approaches to workplace safety before 1974, such as safety management, also informed the Robens philosophy. Safety management as a practice and profession emerged in many British workplaces following the First World War, when it became promoted by organisations such as the Royal Society for the Prevention of Accidents (RoSPA) and later, the British Safety Council. The aim of safety management was to prevent industrial accidents by promoting safety as part of the everyday running of business, for example by promoting training schemes, safety committees and the use of professional safety officers.^35^ As described below, while these approaches were not new, they became increasingly central to tackling workplace accidents in the 1960s, as inspectors and policy makers grew more convinced that the existing regulatory approach, based on an ‘ever-expanding body of legal regulations’, had faltered.^36^

Certainly, by the end of the 1960s inspectors and policy makers believed there was a growing mismatch between the kinds of response they thought was necessary to confront accidents, and the kinds of response required by legislation. Existing health and safety law focused on the physical conditions and hazards of work, as opposed to the social or organisational factors that scientific models and research increasingly showed to be at the root of accidents.^37^ The Factories Act 1961, for instance, laid down detailed minimum requirements for such matters as temperature and ventilation in the workplace, but had nothing to say about such matters as the on-going monitoring of safety performance.^38^

Despite this ‘physical’ emphasis, by the early 1960s inspectors were outspoken about social and organisational factors, calling for arrangements to encourage safe and healthy systems of work and for workers to participate in safety management. Trade unions and safety charities demanded that safety organisations should be established at workplace and industry level as a structured response to the accident problem. Contemporary economic and political developments amplified these concerns, placing them at the heart of the Robens Committee's proposals for ‘a more effectively self-regulating system’.^39^

## Safety Consciousness and Industrial Self-help

This movement in regulatory attention from the ‘physical’ to ‘social’ environment of the workplace was not a new phenomenon in the early 1960s, but had been on-going for several decades. Not least, the continuing development of the safety profession, spearheaded by organisations such as RoSPA, increased the profile of safety management in many firms.^40^ As early as 1927, the government encouraged the setting up of works safety committees, bringing together worker and management representatives to discuss safety problems.^41^ In 1956 the National Joint Advisory Council, a tripartite body bringing together trade union and employer representatives to advise the Minister of Labour on industrial relations, stressed the importance of safety organisation and demanded an increase in safety committees.^42^ These developments urged industry to take greater responsibility for safety and take proactive steps to prevent accidents, instead of addressing hazards after an accident had occurred or relying on reactive statutory intervention (e.g. inspection). Despite these approaches, in 1961 the Chief Inspector of Factories, T. W. McCullough, painted a grim picture of industrial safety organisation:

Too many firms still have no safety organisation whatever, or where it exists it is ineffectual. … Many employers appear to rely on H.M. Inspectors to deal with the safety problems in their works. Inspectors are, of course, always ready to give advice on the best means of promoting safety and health, but responsibility in these matters rests on the occupier. Only through better realisation of that responsibility leading in turn to better safety organisation at the place of work … is substantial progress to be expected.^43^

A sharp rise in industrial accidents had by this point called into question industry's commitment to accident prevention. Reversing the downward trend of previous years, the number of reported accidents under the Factories Act increased by 15 per cent between 1958 and 1961.^44^ The number of accidents suffered by young persons was particular grounds for concern to factory inspectors, suggesting many employers were neglecting their duty to train and supervise new entrants to the workplace—an essential requirement at a time when British productivity was falling (see below).^45^ While the overall number of *fatal* accidents fell, this sudden increase generated significant political attention both within and outside the British government.

In the ‘industrial self-help campaign’, the British government launched a drive to encourage industry to develop safety organisation. One element of this campaign was the MOL's decision to co-operate with the TUC and BEC in developing central accident prevention organisations in industries where they were absent, such as shipbuilding.^46^ The ostensible benefit of such organisations was their help in collating accident statistics, providing guidance material tailored to the industry, and operating training schemes.^47^ Other forms of safety organisation encouraged by the government included industrial health and hygiene services, workers' safety representatives and professional safety officers. From 1965, the government funded RoSPA to help develop safety organisation on a regional basis throughout Britain.^48^

These interventions were framed by the British government as part of a campaign to inculcate ‘safety consciousness’ in industry. McCullough summed up its paternalistic basis in 1963 when he wrote, ‘Safety consciousness … is a form of foresight or alertness, a quality of mind which has to be developed and nurtured.’^49^ Hence, the British government and its inspectors relied upon strategies such as education, advice and persuasion to encourage ‘self-help’, delivered through a range of media including face-to-face advice, safety posters, publications, exhibitions and conferences. One such conference was the joint conference on industrial safety organised by the TUC and BEC in November 1962, which precipitated several joint efforts to stimulate safety organisation over the decade.^50^ The Industrial Health and Safety Centre on Horseferry Road, London, originally opened in 1927 as the Home Office Industrial Museum, also served as a forum for the education of visitors until financial constraints forced its closure in 1980.^51^

Importantly, these various interventions did not stem from any fundamental desire by the government to legislate, although the sluggish response of industry to the call for safety committees later prompted the Labour government to try putting them on a statutory footing. Rather, taking their cue from the conciliatory enforcement philosophy of the Factory Inspectorate, and the ‘voluntarist’ stance of the 1960s Conservative and Labour governments towards industrial relations, they were driven by a desire to help industry meet its obligations. Committed to collective bargaining with a minimum of statutory interference, the TUC also felt that safety organisation was best pursued by voluntary means. While its Social Insurance and Industrial Welfare Committee was keen that the government should boost the number of factory inspectors and strengthen the law, it also accepted that ‘legislation [alone] could not invariably prevent accidents and that it was very necessary to educate people to work safely.’^52^

The industrial self-help campaign was based upon an implicit belief about occupational accidents. Factory inspectors and others in the safety movement had long considered there to be a ‘human factor’ to such accidents, an idea that was encapsulated in contemporary scientific models of accident causation.^53^ At the extreme, this idea was reflected in the psychological construct of ‘accident proneness’ that crystallised in the 1920s and 1930s.^54^ However, by the early 1960s, British factory inspectors accepted that the vast majority of accidents, especially those within the so-called ‘Big Five’, included an intrinsic ‘human’ dimension that resisted legislative control. The ‘Big Five’, accident statistics revealed, included accidents resulting from manual handling, the use of hand tools, falls from heights, strikes against objects and strikes from falling objects. In 1962, they accounted for almost two-thirds of all reported factory accidents.^55^ As the Chief Inspector of Factories, R. K. Christy observed in 1964,

While a proportion of the ‘Big Five’ accidents may be connected with breaches of factory legislation, experience has shown that the majority occur in circumstances which cannot readily be controlled by legislation, for example lack of attention to good industrial housekeeping. … The errors arising from human behaviour unlike the requirement to fence a dangerous machine do not, except to a very limited extent, lend themselves to control by legislation.^56^

**Fig. 1:**
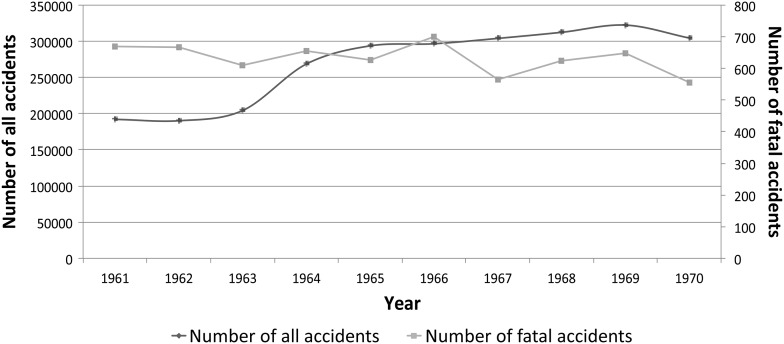
Work accidents in Great Britain, 1961–1970, in premises subject to the Factories Act 1961.

*Source:* Robens, *Safety and Health at Work*, 161–2.

John Burnham has argued that the psychological concept of ‘accident proneness’ receded into the popular imagination by the 1960s, caught between the rise of the new epidemiology on the one hand (the concept of aggregating individuals into risk groups), and safety engineering on the other (the use of physical controls such as machine guards to prevent accidents for entire groups of workers).^57^ Among British factory inspectors, however, engineering approaches to workplace safety were in themselves being called into question around this time. Revised models of accident causation emerging from insurance, such as Frank E. Bird's ‘total loss control’, showed that the accident was the tip of the iceberg of wider managerial or organisational failings in the firm.^58^ Over the 1960s, the idea that certain types of accident remained stubbornly resistant to legislation fuelled growing suspicions among inspectors that first, the vast majority of accidents preventable by engineering means had already been prevented, and second, that the prescriptive legislation outlining these measures was suffering from diminishing returns.^59^ With these diminishing returns, inspectors claimed, the solution to accidents was not more regulation, more inspectors or more enforcement, but a more concerted effort by industry itself. As the Chief Inspector, Bryan Harvey argued, ‘Some of the traditional hazards of the physical environment have been brought under control over the past years. What we must now increasingly tackle is the social or management environment which may underlie poor safety performance.’^60^ Sitting in the early 1970s, the Robens Committee was strongly influenced by this idea of a ‘safety plateau’, suggesting that the future emphasis of the state should not be the further proliferation of regulation, but a renewed focus on encouraging and coordinating voluntary effort.^61^

An even greater spike in the number of recorded accidents under the Factories Act in 1964, however, forced the Labour government and TUC to reappraise this strategy for promoting safety. The total number of 268,648 accidents reported that year represented an increase of almost a third above 1963, and the highest reported figure since the Second World War (Figure 1).^62^ While the causes of this increase were unknown—improved reporting since the start of the industrial self-help campaign, an upswing in industrial production, and the severe winter of 1962–63 all contributed—the Parliamentary Secretary to the Ministry of Labour, Ernest Thornton, was forced to conclude ‘[I]t is reform we need—a new spirit of determination throughout the country to stop the human suffering and waste of our scarce manpower resources which these appalling accident figures represent.’^63^ He added, ‘I think we must appreciate that accidents of this kind cannot easily be reached by legislation, or prevented by factory inspectors. They can, however, be prevented if management and workers develop an active safety consciousness.’^64^

This new and more worrying rise in accidents followed reports suggesting that a significant section of industry had either wilfully ignored, or was unaware of, its statutory obligation to report accidents. In the early 1960s, the government observed that claims to industrial injuries benefit outweighed the number of non-fatal accidents reported to the Factory Inspectorate.^65^ To evaluate this discrepancy, in October 1962 the MOL, in conjunction with the Ministry of Pensions and National Insurance, carried out a survey comparing the level of reported accidents under the Factories Act with claims for industrial injuries benefit, adjusting for accidents that were not reportable. The survey revealed that less than 60 per cent of all notifiable accidents were being reported.^66^ A follow-up enquiry two years later revealed that despite efforts in the interim, such as the sending of a flyer to factory occupiers, industry had made little progress. On average, across the Factories Act, 40 per cent of all accidents were never notified. In smaller factories, those employing fewer than 100 people, a shocking 50 per cent of all accidents were never notified to the government: these premises employed a third of Britain's factory workforce.^67^

The sheer level of under-reporting exposed by the surveys revealed that despite the self-help campaign, many employers were still uninformed about their duties. This neglect of safety was not confined to accident reporting: as previously mentioned, since 1956 the MOL, in association with the TUC and other organisations, had attempted to increase the number of safety committees in firms across the country. Here too, a survey in 1964 revealed that industry's promises could not be relied upon: the number of safety committees in the largest, and supposedly better organised firms had actually decreased since 1956, rather than increased.^68^

It was at this point that the TUC, which had previously supported the voluntary establishment of safety committees, called for their statutory compulsion in a 1964 resolution.^69^ The BEC, however, opposed any such question. While they were open to joint consultation between workers and management in principle, they argued that imposing a legal requirement would undermine voluntary efforts already underway, and encourage employers to meet, rather than exceed, legal requirements.^70^ This was a line the BEC's successor, the CBI, repeated to the Robens Committee.^71^ Examining the previous assurances made by industry, however, the MOL believed that industry's commitment was ‘open to serious doubt’.^72^ During a Parliamentary question on works safety committees in 1966, the MOL warned that

Progress in the past has … been extremely disappointing. … [U]nless there is satisfactory progress over the next few years in the setting up of joint works safety committees on a voluntary basis, [the Minister of Labour] will feel obliged, when the next major revision of the Factories Act takes place, to seek power to require the establishment of machinery for joint consultation in appropriate cases.^73^

In response to this threat, the number of safety committees subsequently increased by 69 per cent, from 5,826 in 1966 to 9,847 in 1969.^74^

That same year, the Employed Persons (Health and Safety) Bill, presented by the Secretary of State for Employment and Productivity, Barbara Castle, attempted to legislate for safety committees in British workplaces.^75^ The Bill provided for recognised trade unions to appoint worker safety representatives in premises where 10 or more persons were employed. In premises where over 100 persons were employed, the employer was required to establish a safety committee if the safety representatives requested. However, this Bill was lost following the dissolution of Parliament in 1970, pending the general election. It was not until 1977, following the HSW Act, that recognised trade unions gained the right to appoint safety representatives, in one of the few significant departures from the Robens Committee's recommendations.^76^

By the end of 1960s, therefore, efforts to improve industrial safety organisation by voluntary means alone had demonstrably failed, requiring the threat of legislation to work. Despite this, the Robens Committee, sitting between 1970 and 1972, did not recommend the further extension of prescriptive legislation, or an increase in prosecution. Instead, it recommended a turn to goal-setting legislation and greater self-regulation by industry. In relation to safety committees, Robens avoided prescribing any particular arrangement, preferring instead a general requirement for employers to consult their workers.^77^ The reason for this turn, as will become apparent, was not only that Robens bought the CBI's argument that legislating for joint consultation would undermine voluntary efforts, but also that his thinking was captured by the conciliatory approach of the Factory Inspectorate and its talk of diminishing regulatory returns.

## Safety, Productivity and Self-help

In addition to accidents, economic and political concerns in the 1960s promoted the movement of regulatory attention onto safety organisation. Fears about Britain's low productivity compared to its competitors, and its increasingly disorderly industrial relations, persuaded the government to intervene ever more closely in work activity. Such concerns helped promote, by 1970, a growing synergy between questions of safety, productivity and self-help in regulatory discourse, one that supported the emergent Robens philosophy.

The 1960s was not the first time that questions of productivity had encouraged the British government to intervene in industrial safety, health and welfare. Historians have written at length about how the militaristic needs of the British state in the First and Second World Wars acted to focus political attention on the needs of the industrial worker. In the Second World War, for example, new orders were made under the Factories Act, introducing requirements for such matters as lighting, canteens and first-aid facilities.^78^

In the 1960s, it was not military competition but global trade that highlighted the economic costs of poor health and safety. Over the decade Britain's share of world trade declined, from around 20 per cent in 1955 to 13 per cent in 1970.^79^ In 1965, around the time accident statistics had called into question industry's commitment to work safety, comparative levels of worker output per capita were 32 per cent higher in West Germany, and a remarkable 84 per cent higher in the USA.^80^ Britain's declining productivity resulted in a growing trade deficit, culminating in Harold Wilson's decision to devalue sterling in November 1967. Within this context, the costs of absenteeism, sickness and injury resulting from industrial accidents and disease became increasingly contentious. In 1967 the number of working days lost per year to occupational accidents and disease was reckoned at 23 million, a figure that was ten times the comparable number lost to strikes, both official and ‘wildcat’.^81^

In an era of full employment and political concerns about inflation, the key to industrial productivity was seen to lie in improving industrial efficiency, of which safety was a core component (thus Thornton's assertion, above, about the ‘waste of … scarce manpower resources’ that the accident figures represented).^82^ Industrial training was one area where safety and productivity needs converged, and over the 1960s the government spent significant effort in attempting to promote safety in the curricula of industrial training schemes.^83^

The synergy between safety, self-help and productivity was also reinforced in industrial relations, which likewise painted a picture of a disorganised and inefficient work environment. Between 1956 and 1966, the number of strikes outside mining increased by 142 per cent.^84^ The growing problem of strikes motivated the 1964–70 Labour government to appoint the Royal Commission on Trade Unions and Employer's Associations (Donovan Commission) in 1965.^85^ Its membership was drawn from both sides of industry and included Lord Robens, then in his position as chairman of the National Coal Board. Its 1968 report concluded that there were ‘two systems’ of industrial relations in Britain, in fundamental conflict with one another. There was the ‘formal system’, comprising official institutions and industry-wide collective agreements, and there was the ‘informal system’, comprising the actual behaviour of shop stewards, managements and others on the ground.^86^ The fundamental problem with British industrial relations, the Commission argued, was that the ‘informal system’ was dominating, and thus undermining, the ‘formal system’.^87^ Their proposed solution, in some respects analogous to the Robens Committee's later recommendations, was statutory intervention to strengthen and support *voluntary* arrangements regulating industrial relations at workplace level. The Donovan Commission included safety as a central objective in its proposals, providing mechanisms for ‘regular joint discussion of measures to promote safety at work’.^88^ Barbara Castle's move to legislate for joint consultation after 1966 was thus intimately woven with this wider trend towards greater statutory intervention in industrial relations.

Robens himself was in a unique position to bring these ideas about safety, productivity and self-help together. As chairman of the National Coal Board, Robens experienced first-hand the shortcomings of the existing regulatory system at Aberfan, and led a nationalised industry with a relatively well-developed approach to safety compared to other industries.^89^ As a member of the Donovan Commission, Robens learnt how disorganised workplaces promoted industrial unrest and undermined national productivity. As a former trade union official, Labour MP and Minister of Labour in the 1950s, Robens was a passionate advocator of industrial safety, recommending that legislation should be extended to offices, shops and other non-industrial workplaces.^90^

In 1970, just before he became chairman of the Committee on Safety and Health at Work, Robens published an insightful book that demonstrates how these considerations informed his regulatory philosophy. In *Human Engineering*, Robens proposed a comprehensive overhaul of British industry, boosting national competitiveness by exploiting ‘the vast potential of the manpower of this country, the native genius and natural initiative’.^91^ Robens cited inefficient management as the overriding explanation for Britain's poor economic performance. The main reason Britain was uncompetitive was because British industry could not efficiently utilise its labour.^92^ Poor safety performance signified a badly managed workplace, where workers had little say in the managerial decisions affecting their work. Arrangements that therefore encouraged worker participation, and facilitated safety as a matter of good business practice, should therefore be strengthened as a matter of explicit government policy. Statutory regulation, in his view, encouraged the notion that responsibility for safety rested with the government, not industry. In an evocative quote, suggesting that Robens thought about redistributing responsibility for safety before the Committee on Safety and Health at Work was even established, he wrote: ‘Not until wise managements recognise the importance of safety at the place of employment as an integral part of efficiency will the requirement for inspectors and enforcement virtually disappear.’^93^ As the preceding analysis has revealed, such an opinion reflected the stance of the bodies responsible for administering safety and health legislation before 1974. Robens now carried these ideas and beliefs into his deliberations on the Committee on Safety and Health at Work.

## The Committee on Safety and Health at Work

The decision to appoint an independent committee to review how Britain's health and safety system operated was taken strategically. In 1967, the Minister of Labour, Ray Gunter, began consultations with trade unions, employers' associations and other bodies on the Ministry's plans to revise and consolidate the Factories Act 1961 and Offices, Shops and Railway Premises Act 1963.^94^ Despite proposals submitted in December 1967, however, which envisaged an Act of a much wider application than previous statutes, as of 1969 there was little agreement between the parties on how to proceed.

Barbara Castle and her colleagues at the Department of Employment and Productivity inherited these proposals when the MOL was dissolved in 1968. Castle believed that the 1967 proposals did not go far enough to remedy the deficiencies of the existing regulatory system, such as its exclusion of many groups of workers and members of the public at risk from work activity. The government too had lost confidence in the proposals, deciding in January 1969 to rule out comprehensive legislation in the 1969–70 Parliamentary session.^95^ Although Castle proceeded with her own interim legislation, the Employed Persons (Health and Safety) Bill, this focused on just two of the many issues requiring attention by the end of the 1960s, joint consultation and proposals for a new Employment Medical Advisory Service. Castle thus appointed a committee largely as a device to break through this impasse. In a note to her colleagues, Castle wrote how a small committee, composed of a handful of members, would be more likely to produce quick results and conclusions palatable to both trade unions and employers.^96^ In a letter to the General Secretary of the TUC, Victor Feather, Castle wrote:

The conclusion I have come to is that the matter can be satisfactorily dealt with only by having a high-level outside enquiry. I have in mind a small body—perhaps a chairman and 3 or 4 members—who would, without going into the detail of the existing legislation, take a general look at the way the present system works right across the field.^97^

The Committee on Safety and Health at Work was duly appointed on 29 May 1970, and had its first meeting on the 23 June. The idea that Robens should be appointed chairman came from Victor Feather, suggesting that Robens retained credibility among the trade union movement despite the reputational damage inflicted by Aberfan.^98^ In addition to Robens, fellow members included the law professor, John Wood; the management consultant, Anne Shaw; the Conservative MP, Mervyn Pike; the radiologist Sir Brian Windeyer; the trade unionist Sydney Robinson and the industrialist, George Beeby.^99^

Between 1970 and 1972, the Committee collected evidence from over 200 individuals and organisations concerned with safety and health at work, including government departments, trade unions, employers' associations and insurers. The Committee embarked on several industrial visits to see how the system worked in practice, and also conducted international visits to familiarise themselves with foreign systems. Between March and September 1971 the Committee visited Sweden, West Germany, Canada and the USA.^100^

The health and safety systems in these countries worked very differently to those in Britain, although each had potential lessons to offer the Committee. In the USA, President Nixon had recently signed the Occupational Safety and Health Act (OSHA) 1970. Designed ‘to assure so far as possible every working man and woman in the Nation safe and healthful working conditions’, OSHA was the first federal foray into occupational safety and health legislation, such legislation having previously been worked out at state level.^101^ As in Britain, OSHA was prompted over concerns about the rising level of industrial accidents: as of 1970, 14,000 American workers were killed at work each year. The Act established a general duty for employers to provide employment free from recognised hazards, and enabled the Secretary of Labor to promulgate standards developed through advisory committees and national standard-making organisations, taking into account the views of interested parties.^102^ The Robens Committee considered the statutory approval of voluntary standards an important regulatory tool, emphasising in its report that further weight should be attached to them in future.^103^

Sweden offered other lessons. There, the Workers' Protection Act had established comprehensive statutory protection against occupational accidents and disease in 1949. The same Act also provided for the appointment of safety representatives and joint safety committees.^104^ Swedish arrangements for joint consultation were looked upon by British trade unions particularly favourably in the 1960s, and were considered a possible model for British arrangements, as industry's commitment to establish safety committees was in question. As a paper for the National Joint Advisory Council's Industrial Safety Sub-Committee remarked, ‘the Swedish example of safety delegates and safety committees at work shop level seems to offer a workable system of compulsory joint consultation with voluntary cooperation super-imposed.’^105^

Throughout its work, the Robens Committee was assisted by a Secretariat composed of seconded civil servants, headed by Matthew Wake of the Department of Employment (DE). The Secretariat played a crucial role in organising the Committee's work, preparing briefing notes, arranging meetings and processing evidence. They acted as a vital link between the Committee, which was essentially a ‘lay’ group of officials from across the political spectrum (or those, like Robens, with limited experience), and those who had ‘on the ground’ experience of safety and health policy or practice. In the early stages of its work, the Committee was also helped by the DE itself, which prepared several background documents to get the Committee underway. The work of the Secretariat and DE in filtering and shaping the Committee's arguments has been little acknowledged by academic studies, which have tended to focus on the published report itself. An analysis of the background documents prepared for the Committee reveal just how influenced the Committee was by the views and beliefs of its sponsoring department.

An early paper, for instance, advanced a view of the regulatory system that was automatically accepted by the Committee, highlighting ‘the multiplicity of enforcing agencies, the multiplicity and overlap of statutes, the distinction between safety and health of employed persons and safety and health of members of the public, [and] gaps in the coverage of the legislation’.^106^ The DE had been preparing comprehensive legislation since 1967, and thus this interpretation gently prodded the Committee toward reforms under the DE's purview. An early review of evidence just six months into the Committee's proceedings, moreover, highlighted the Factory Inspectorate's belief that ‘the existence of a mass of detailed restrictive legislation may inhibit the natural development of self-help and continuous self-regulation by industry itself’.^107^ This was virtually identical to the Robens Committee's eventual assertion, ‘the existence of such a mass of law has an unfortunate and all-pervading psychological effect. People are heavily conditioned to think of safety and health at work as in the first and most important instance a matter of detailed rules imposed by external agencies.’^108^

Oral evidence presented to the Committee also suggests a cognitive bias—if unconscious—towards the views of the DE and Factory Inspectorate. One of the first presentations delivered to the Committee was from the Chief Inspector of Factories, John Plumbe, who repeated his assertion that the Inspectorate's enforcement work was subject to diminishing returns.^109^ An increase in prosecution, Plumbe suggested, would be counter-constructive to health and safety, since it would ‘reduce the “public image impact” of prosecution action’. Instead, the Inspectorate considered the law ‘a powerful reinforcement of their persuasive functions … one to be kept in the background and used as last resort’.^110^ This belief, of huge importance to the subsequent enforcement of British health and safety regulation, received no critical scrutiny from the Robens Committee and found its way directly into its report. The idea that excessive legislation deterred or undermined individual responsibility, of course, was a position entirely in agreement with Robens' expressed ethos in *Human Engineering*.

On the other hand, the Committee appears to have quickly dismissed the TUC's view that the government needed to devote more resources to accident prevention, increasing both the number of factory inspectors and the level of fines imposed in court. In his oral evidence, C. R. Dale, the Secretary of the TUC's Social Insurance and Industrial Welfare Committee, argued for a continuation of detailed, specific laws, because general requirements were harder to enforce. The question for the TUC was not whether the balance between statutory and voluntary effort was correct, but whether they could be *sustained*: in his view, there was neither sufficient enforcement of the law, nor too many regulations.^111^ In contrast, the Robens Committee argued that this view, based upon ‘an ever-expanding body of legal regulations enforced by an ever-increasing army of inspectors’, was no longer tenable.^112^

Except for joint consultation, the Robens Committee was more sympathetic to CBI proposals, which were more in line with DE and Factory Inspectorate proposals as they had developed from 1967.^113^ First, the CBI argued that the proliferation of health and safety law over time had obscured the common law duty of care; there needed to be an adjustment in legislation to emphasise general duties. As a briefing paper submitted to the CBI's working party on the Robens Committee suggested,

What is wanted is not just new legislation but a completely new approach and method of presentation centred upon the predominance of the basic common law principle which places responsibility on every individual for reasonable conduct in his relationship with others.^114^

Second, the CBI concurred with the DE's view that the growing mass of law was becoming unintelligible, meriting urgent rationalisation. The CBI believed that an ‘enabling’ Act expressing general principles would be more comprehensible to employers, with subordinate regulations dealing with specific matters. On safety committees, the CBI maintained that statutory compulsion was unlikely to work, since it depended upon ‘a positive desire on both sides of industry to work together for common objectives’.^115^ Indeed, CBI representatives to the Robens Committee argued that ‘“compulsory joint consultation” was a contradiction in terms’.^116^

The CBI's oral evidence, alongside that of other parties, helps illuminate the initial considerations that informed the Robens Committee. They are more immediately revealing than the minutes of the Committee itself, which tend to obscure points of contestation between members, and concentrate on logistical matters, such as upcoming visits. However, the minutes show that the Committee reached some of its most important decisions relatively early in its proceedings. By January 1971, the Committee had already determined there should be a new, comprehensive enactment applying to all employees, and there should be renewed focus on better attitudes and responsibility at work; there were limits to what legislation alone could achieve.^117^

The Committee's report was published in July 1972, and brought together the various ideas about accidents, regulation and the role of the state discussed so far. Its fundamental argument was that the existing statutory and voluntary approach to safety and health at work had reached its limit. The defects of the existing system were revealed not only by a host of legislative and administrative problems, but also a disgraceful ‘humanitarian’ cost of 1,000 fatalities and half a million injuries a year.^118^

The Committee portrayed a bloated, fragmented, reactive and overly prescriptive system, one that was outmoded and in urgent need of rationalisation. Not only had the existing framework reached the limit of its comprehensibility to industry and government, but it also undermined the initiative and ‘safety consciousness’ inspectors were trying to instil. It was in this context that the Committee advanced its contentious argument that ‘the most important single reason for accidents at work is apathy.’^119^

The Robens Committee accepted without reservation the Factory Inspectorate's view that the existing statutory approach was becoming ‘counter-productive’.^120^ From this perspective, the further extension of detailed health and safety law was no longer workable: it could not keep abreast of changes of industry and technology, and rapidly became obsolete. These considerations lent support to the Committee's primary conclusion, which was that ‘*there are severe practical limits on the extent to which progressively better standards of safety and health at work can be brought about through negative regulation by external agencies. We need a more effectively self-regulating system.*’^121^

The Committee's proposed solution to this problem was a wholesale redistribution of responsibility away from statutory regulation, to ‘*those who create the risks and those who work with them*.’^122^ In furtherance of this aim, informed by CBI and DE proposals, Robens argued that the substance of the existing law should be revised and reconfigured under the aegis of a single Act, applying to all workers and workplaces. This Act would express the general principles of safety and health, but leave specifics, such as requirements for particular industries and hazards, to subordinate regulations and codes of practice.^123^ While regulations (including prescriptive regulations imposing absolute duties) would still remain as part of a suite of tools at the disposal of regulators, the emphasis was squarely on promoting safety and health by non-statutory means such as codes of practice and guidance.^124^

Corresponding with this programme of legislative reform, Robens argued that the efficiency of statutory regulation could be increased by unifying the various government departments and inspectorates responsible for health and safety under one institution, the National Authority for Safety and Health at Work. This Authority would be self-contained, hived off from central government, and managed by a board composed of representatives of trade unions, employers' associations and other ‘user interests’.^125^ By reallocating responsibility away from the government to those groups with direct experience of industry and working conditions, Robens institutionalised his philosophy of self-regulation in the management of the new bodies established following the HSW Act.

On enforcement, Robens' recommendations strongly reflected the Factory Inspectorate's belief that legislation ‘should seek to promote, as much as to control’.^126^ Robens advised against the use of prosecution for most offences under health and safety law, preferring instead the use of new administrative sanctions, improvement and prohibition notices, to encourage good practice. Indeed, the Robens Committee believed the role of the state was to facilitate good practice, establishing and strengthening the arrangements through which voluntary effort, or ‘self-regulation’ could thrive.

The Committee's language clearly evoked the contemporary concerns about productivity and poor industrial relations, reflecting the same notions of a disordered and inefficient work environment Robens had expressed in *Human Engineering*. Arrangements such as written safety policies were integral to raising standards, allocating responsibility and introducing a considered, scientific approach to accident prevention. Occupational safety and health, the Committee asserted, was ‘an essential feature of good management’ and needed to be treated as a ‘normal management function’, in much the same way as marketing or production. An efficient workplace was one where everyone, from the boardroom to shop floor, understood and carried out their responsibilities.^127^

This idea was intimately bound with the established voluntarist model of industrial relations, which conceptualised an open and on-going dialogue between ‘capital’ and ‘labour’, in which the state did not directly intervene. Through this dialogue, it was assumed, accident prevention would be afforded a higher, if not equivalent priority to other items on the business or industrial relations agenda. This concept became encapsulated in the Committee's controversial remark that ‘There is a greater natural identity of interest between “the two sides” [of industry] in relation to safety and health problems than in most other matters. There is no legitimate scope for “bargaining” on safety and health issues’.^128^

The Robens Report was supported by an extensive publicity campaign, in which Lord Robens took to the television, newspapers and radio to explain his recommendations. Overall, both sides of industry and the two main political parties welcomed the report. While the TUC and CBI disagreed with the report's stance on specific issues, such as the form and content of new regulation, they felt that the report represented an advance over the existing system. Writing for the Amalgamated Union of Engineering Workers, for example, Victor Feather argued that the Robens Report was an important step forward, and history needed to remember its publication.^129^ The TUC and CBI were also willing to overlook their differences to lobby for Robens' proposals to be speedily enacted: following the report's publication, the TUC and CBI wrote a joint letter to Employment Secretary, Maurice Macmillan, urging him to bring forward legislation as soon as possible.^130^ In the same way, although the Labour and Conservative parties expressed differences over the role of trade unions in appointing safety representatives, the HSW Act received bipartisan support in Parliament and was passed by an incoming Labour government in July 1974 with only minor changes to a Bill presented by the Conservative Employment Secretary, William Whitelaw, in January 1974.^131^

Nevertheless, the report received various criticisms in print and private. Commentators such as Patrick Kinnersly, as we have seen, attacked Robens' emphasis on ‘apathy’ and his notion of an ‘identity of interest’ between employers and workers, that safety could somehow be divorced from the exigencies of industrial relations.^132^ The *Guardian* reported that by emphasising self-regulation, Robens placed ‘too much faith in human nature’.^133^ The Labour backbencher Neil Kinnock scoffed that the Robens philosophy effectively meant ‘If we have less law, we shall have more safety.’^134^ Others, such as James Tye of the British Safety Council, complained that the report had received little exposure at all.^135^ Other newspapers, however, took a more generous view, with the *Daily Mirror* reporting how it formed ‘a real drive to improve [Britain's] shaming record of human suffering and economic loss’.^136^ What is apparent is that while contemporary criticisms against the Robens Report were expressed, they were relatively few in number, and directed at specific aspects of the Report rather than its overall recommendations. They do not appear to have influenced the subsequent passage of the HSW Act in any meaningful way.

Instead, the Robens Report seems to have generated significant administrative problems for Whitehall, with the Labour Employment Secretary Michael Foot remarking, on introducing the HSW Bill in 1974, that the report produced ‘a prolonged and intensive period of interdepartmental consultation’.^137^ These problems were less a reaction to the Report's emphasis on self-regulation than a response to its unifying approach and implications for the machinery of government.^138^ Several departments, in particular the Department of the Environment, resisted the demand that their safety and health functions should be devolved to a new quasi-independent authority. There were also questions about the status of inspectors and policy makers transferred to the agency: whether they would be independent of private interests, and whether they would continue to be civil servants. Fleshing out Robens' proposals between 1972 and 1974, officials ultimately created two new agencies instead of the single national authority envisaged by Robens.^139^ The Health and Safety Commission (HSC) incorporated trade union, employer and public interests in the development of national health and safety policy, and were crown appointees, while officials working for the Health and Safety Executive (HSE) continued to be civil servants, enforcing the law, undertaking research and publicity, and providing advice to the HSC. This institutional separation between interest-based policy making and enforcement continued in force until 2008, when the HSC effectively became the management board of the new unified HSE.

## Conclusion

Despite the ascendancy of European health and safety law in recent decades, the HSW Act continues to provide the legislative foundation for health and safety regulation in Britain to this day, over 40 years after it was passed. Thus, the self-regulatory philosophy that crystallised in the 1960s and early 1970s, and propagated by the Robens Committee, continues to provide the conceptual framework within which occupational accidents and diseases are visualised, legislation is viewed and the role of the state expressed. This is remarkable considering the extraordinary changes that have occurred in the British labour market and economic and political landscape since the 1960s, notably the decline in manufacturing. To many in government and health and safety regulation, this longevity is testament to the flexibility that underpins the ‘goal oriented’ and ‘risk based’ HSW Act.^140^ The system Robens envisioned allows the law to be revised relatively quickly, in comparison with before 1974, and keep abreast of technological and industrial change.

However, it is also remarkable that recent government reports, seeking to reduce the ‘burdens’ of health and safety regulation on business and operating within a deregulatory context that has existed since at least the 1980s, have sought to recapture some of the original spirit of the Robens philosophy. In some respects, the report *Reclaiming Health and Safety for All*, presented by the Professor of Risk Management, Ragnar Löfstedt, in 2011, was no more than a restatement of the system's founding principles.^141^

By laying bare the principles that underpin the HSW Act, this article has provided a foundation for the historical study of health and safety in Britain after 1974. Since the HSW Act structures and facilitates voluntary effort on the part of employers and workers, these ideas and assumptions need to be acknowledged for this voluntary effort, or ‘self-regulation’, to be understood. This article therefore complements studies focusing on the behaviours and attitudes of employers and workers in particular industries and workplaces.

However, this article has also shown how the post-1974 history of health and safety cannot be separated from earlier efforts to prevent accidents and disease in the workplace: the principles underpinning the current regulatory framework need to be considered part of a longer evolutionary process, in terms of a regulatory system that has developed since the early nineteenth century. While many of these principles were inherent to the regulatory philosophy of the pre-1974 Factory Inspectorate, the social, political and economic context of the 1960s provided a unique set of circumstances that placed them at the heart of the Robens Committee's agenda: concerns about the incidence of accidents, productivity, industrial relations and the diminishing returns of statutory effort. As such, the current framework of health and safety regulation in Britain continues to embody a particular vision of regulation laid down at a particular moment, over 40 years ago. Health and safety may appear a modern phenomenon, but in its contours—its shape, structure and underlying ideas—it is distinctly middle-aged.

## Funding

This work was supported by the Economic and Social Research Council [grant number ES/J500021/1].
